# Population and sexual fluctuation of Calliphoridae and Mesembrinellidae (Diptera: Oestroidea) in the Atlantic forest of Rio de Janeiro

**DOI:** 10.1371/journal.pone.0318496

**Published:** 2025-04-21

**Authors:** Mariana dos Passos Nunes, Wellington Thadeu de Alcantara Azevedo, Alexandre Sousa da Silva, Jeronimo Alencar, Cláudia Soares Santos Lessa, Valéria Magalhães Aguiar

**Affiliations:** 1 Departamento de Microbiologia e Parasitologia, Instituto Biomédico, Centro de Ciências Biológicas e da Saúde, Universidade Federal do Estado do Rio de Janeiro, Rio de Janeiro, Brazil; 2 Programa de Pós-Graduação em Ciências Biológicas, Instituto de Biociências, Universidade Federal do Estado do Rio de Janeiro, Rio de Janeiro, Brazil; 3 Programa de Pós-Graduação Biologia Animal, Instituto de Ciências Biológicas e da Saúde, Universidade Federal Rural do Rio de Janeiro, Seropédica, Brazil; 4 Departamento de Matemática e Estatística, Instituto de Biociências, Centro de Ciências Biológicas e da Saúde, Universidade Federal do Estado do Rio de Janeiro, Rio de Janeiro, Brazil; 5 Laboratório de Diptera, Fundação Oswaldo Cruz, Rio de Janeiro, Brazil; Universidade Estadual Paulista: Universidade Estadual Paulista Julio de Mesquita Filho, BRAZIL

## Abstract

Dipterans of the Calliphoridae and Mesembrinellidae families are of high relevance in the Atlantic Forest of Rio de Janeiro, and it is important to examine their diversity and abundance in the different ecological areas of this biome over a time interval. This study aimed to study the diversity and abundance of Calliphoridae and Mesembrinellidae by evaluating the sexual variation and the influence of abiotic factors (average temperature, relative humidity and total precipitation) on the capture of insects collected during the four seasons of the year. Four traps were installed in each ecological area containing 300 grams of beef liver as attractive bait, which remained exposed for 48 hours in each season during the period between autumn 2021 and summer 2022. The collected dipterans were sacrificed, sent to the Laboratório de Estudos de Dípteros (LED-UNIRIO), and taxonomically identified. The Kruskal-Wallis and Wilcoxon tests were used to examine the influence of the four seasons on the abundance, and the Spearman correlation was used to relate abundance to abiotic variables. A total of 2,826 dipterans were collected during the four seasons of the year, represented by nine species of the Calliphoridae family and ten of the Mesembrinellidae family. During the summer, a numerically larger amount of insects was collected, but the Kruskal-Wallis test (chi-square = 5.2781, p = 0.1525) showed there was no significant difference between the abundance of the species collected and the seasons. Spearman’s correlation showed that most species did not show a significant correlation between their respective abundances and the analyzed abiotic factors. The Wilcoxon test indicated that there is a significant difference between the abundance of females and males, with females being significantly more abundant than males, however the difference is statistically greater within the Calliphoridae family (W = 60.49, p = 5.8x10^-12^) in relation to the Mesembrinellidae family (W = 1231.5, p = 0.019).

## Introduction

Tropical biomes are renowned for their high biodiversity and complex ecological networks with diverse niches and habitats. Among these biomes, the Brazilian Atlantic Forest is particularly notable, encompassing various ecosystems such as sandbanks, mangroves, high-altitude fields, and a wide array of forest formations. The city and state of Rio de Janeiro are part of this biome, which is considered one of the richest in biodiversity in the world and the most threatened in Brazil [1]. Being an important biodiversity hotspot, it covers approximately 13% of the Brazilian territory and, with a humid tropical climate, has high temperatures and relative humidity of the air (average 25 ºC and 80%, respectively), in addition to a high rainfall volume [2].

Due to the gradual and disorderly expansion of urban areas that negatively impacts habitats, it is of fundamental relevance to know the organisms that play an ecological role in the cycling of nutrients in the different ecosystems to develop strategies and measures that protect this fauna and, consequently, its habitats [3].Within the fauna responsible for decomposing organic matter and recycling nutrients, various groups of arthropods stand out, including several species of the order Diptera. These highly diverse and abundant organisms play a crucial role in decomposing various organic materials, including putrefying flesh, animal carcasses, manure, fungi, and decaying fruits [4,5].

Several factors can influence dipteran communities, such as resource availability and the type of environment, which includes forested, urban, and intermediate habitats; and additionally, environmental factors such as temperature, relative humidity, rainfall also play a role; potentially affecting dipteran communities both positively and negatively [6]. As the necrophagous dipterans have the habit of landing on decomposing organic matter such as excrement, urban garbage and carcasses, as well as living in the intra-household environment, they have wide medical-sanitary, ecological, and forensic importance. They are identified as carriers of enteropathogenic agents, such as viruses, bacteria, protozoan cysts, and trematode eggs, cestodes and nematodes, in addition to being able to act as ectoparasites, endoparasites, and/or parasitoids of mammals or arthropods during their immature stages [7].

Among the most abundant dipterans in Brazil, as well as in the state of Rio de Janeiro, are the families Calliphoridae, which is particularly important due to its wide global distribution, and Mesembrinellidae, which is notable for its exclusive distribution in the Neotropical region [8], belonging to the super family Oestroidea, which in turn belongs to the clades Brachycera, Cyclorrhapha and Calyptratae. For calliphorids, especially females, it is of fundamental importance to quickly find resources, such as a carcass, as their reproduction depends on it [9,10]. For Mesembrinellidae species, larval development occurs inside the maternal oviduct, and the maggots are released to the external environment only when they reach the last instar, when they are about to pupate. This characteristic where the female does not oviposit is called adenotrophic viviparity [11]. As dipterans of high interest in the Atlantic Forest, being easy to sample and showing quick responses to environmental changes caused by humans at the population level, they are good bioindicators [12]. Therefore, it is important to evaluate their diversity and abundance across different ecological areas of this biome over a temporal gradient.

## Objectives

This study aimed to assess the diversity and abundance of dipteran species from the families Calliphoridae and Mesembrinellidae collected during the four seasons of the year between 2021 and 2022, in different ecological areas of the state of Rio de Janeiro. Additionally, it aimed to analyze whether there is a relationship between abundance, the proportion of males to females, and abiotic factors such as temperature, relative humidity, and total rainfall on the capture of insects.

## Materials and methods

**Fig 1:**
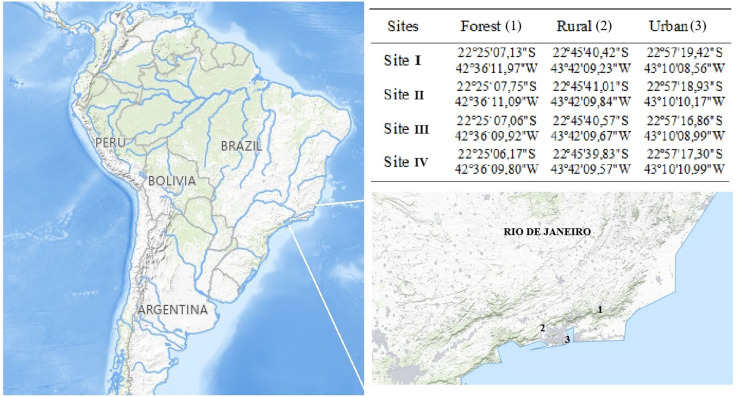
Mage illustrating the three ecological areas in the state of Rio de Janeiro where the research was conducted : (1) forest area at Parque Estadual dos Três Picos; (2) rural area at the cattle farming site on the campus of Universidade Federal Rural do Rio de Janeiro; and (3) urban area on the campus of Universidade Federal do Estado do Rio de Janeiro, Urca. Four traps were installed at georeferenced sites (labeled as site I, site II, site III, and site IV) in each of these three ecological areas. Source: USGS National Map Viewer.

The collections took place at four geographically referenced sites across three ecological areas studied in the State of Rio de Janeiro according to what is shown in Fig 1, where a trap was set at each point to capture Diptera. These three areas correspond to the Parque Estadual dos Três Picos (PETP), a Conservation Unit located in the mountainous region of the state, with Scientific Research Authorization in the Conservation Unit of the INEA n° 019/2020; a rural area located at Universidade Federal Rural do Rio de Janeiro (UFRRJ) in the municipality of Seropédica; and the third area selected for this study, the Universidade Federal do Estado do Rio de Janeiro (UNIRIO), campus located in the neighborhood of Urca, in the city of Rio de Janeiro. The experiment was conducted from autumn 2021 to summer 2022. In each ecological area analyzed, four traps made using PVC pipes were placed, as described by Mello et al. [13].

Each of these three ecological areas was chosen to best represent forest, rural, and urban environments. Within these areas, four specific collection points were selected, approximately 100 meters apart, with one trap installed at each point. The traps, each containing 300 grams of beef liver as bait effective for capturing blowflies [14], were left exposed for 48 hours on tree branches 1.5 meters above the ground. This process was repeated in each ecological area during each of the four seasons, resulting in a total of 12 traps per season. The sampling sessions for the three ecological areas were scheduled to occur within the same week in each season (autumn, winter, spring, and summer) with the aim of obtaining data on the abundance of dipterans in response to different abiotic variables (temperature, humidity, and precipitation), ensuring comparable data across areas and periods. The collection points were carefully chosen to reflect the characteristic conditions of each ecological area and season, minimizing potential biases related to environmental variability and ensuring robust analysis of the collected data.

Temperature, humidity, and total precipitation were recorded using data collected from the nearest official meteorological stations to each sampling point during all collection periods. The three stations used were Nova Friburgo - Salinas (A624), located 10 km from the sampling points in the forest area; Seropédica - Ecologia Agrícola (A601), located 2 km from the sampling points in the rural area; and Rio de Janeiro - Forte de Copacabana (A652), located 3 km from the sampling points in the urban area. All captured dipterans were sacrificed in a solution based on ethyl alcohol and ethyl acetate. They were then led to the Laboratório de Estudos Diptera (LED-UNIRIO), where they were placed with their respective identification to Petri dishes lined with absorbent paper, sealed with PVC film and stored in a freezer at -4°C until the sorting of insects of the families Calliphoridae and Mesembrinellidae, according to the methodology already described [15]. The insects were then identified under incident light using stereoscopic microscopes (Olympus SZX7), following the taxonomic keys of Kosmann [6] and Mello et al. [16] affixed and stored in the entomological collection of the National Museum and the LED-UNIRIO collection.

The Shapiro-Wilk normality test was used to evaluate the abundance variable, and, as there was no normality in the data, nonparametric tests were applied, such as the Kruskal-Wallis and Wilcoxon tests, which compare independent samples. They were used in order to examine the fluctuation of abundance in the four seasons of the year, and also to analyze the proportion of males to females individuals collected from the two families, evaluating the degree of association between these variables, considering a significance level of 5% for the tests [17,18]. All statistics were performed using R version 4.2.1.

The Spearman correlation was used to relate the abundance with the abiotic variables (temperature, relative humidity and rainfall). Graphs were generated showing the coefficient of this correlation, where the values can vary within the range of -1 to 1. A more positive value indicates a positive correlation, while a more negative value indicates an inverse correlation.

## Results

**Fig 2:**
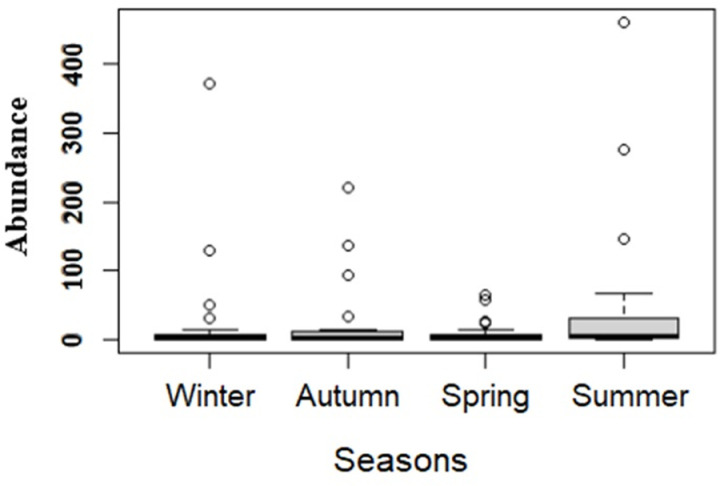
*Box plot* of the abundance of Calliphoridae and Mesembrinellidae in the four collection seasons summing the three collection areas: forest area (Parque Estadual dos Três Picos- Cachoeiras de Macacu), rural area (UFRRJ- Seropédica) and urban area (UNIRIO, campus Urca- RJ).

A total of 2826 dipterans were collected during the period from autumn 2021 to summer 2022, represented by 9 species from the Calliphoridae family, with 2522 specimens accounting for 89.24%, and 10 species from the Mesembrinellidae family, with 304 specimens and 10.76% of the total. During the summer a numerically larger number of insects were collected when compared to the other seasons, and in the spring, a relatively smaller amount was collected, as shown in Table 1 and Fig 2. However, it was verified by the Kruskal-Wallis test (chi-square = 5.2781, p = 0.1525) that there was no significant difference between abundance and seasons.

**Table 1:** Abundance of Calliphoridae and Mesembrinellidae species collected in each of the four seasons of the year, summing the three environments: forest area (Parque Estadual dos Três Picos - Cachoeiras de Macacu), rural area (UFRRJ - Seropédica), and urban area (UNIRIO, Urca campus - RJ), between 2021 and 2022.

Taxa	Autumn	Winter	Spring	Summer	Total
CALLIPHORIDAE					
*Chrysomya albiceps* Wiedemann, 1819	–	3	–	1	4
*Chrysomya megacephala* Fabricius, 1794	–	8	–	19	27
*Cochliomyia hominivorax* Coquerel, 1858	–	18	2	3	23
*Hemilucilia benoisti* Séguy, 1925	3	–	–	1	4
*Hemilucilia segmentaria* Fabricius, 1805	232	132	37	161	562
*Hemilucilia semidiaphana* Rondani, 1850	24	14	4	212	254
*Lucilia cuprina* Wiedemann, 1830	2		1	9	12
*Lucilia eximia* Wiedemann, 1819	262	393	179	782	1,616
*Paralucilia nigrofacialis* Mello, 1969	–	–	–	20	20
MESEMBRINELLIDAE					
*Laneela nigripes* Guimarães, 1977	44	49	21	61	175
*Mesembrinella bellardiana* Aldrich, 1922	48	31	13	9	101
*Mesembrinella peregrina* Aldrich, 1922	–	5	–	–	5
*Mesembrinella semihyalina* Mello, 1967	2	–	–	1	3
*Mesembrinella currani* Guimarães, 1977	2	–	1	–	3
*Eumesembrinella quadrilineata* Fabricius, 1805	3	2	3	–	8
*Eumesembrinella cyaneicyncta* Surcouf, 1919		2			2
*Eumesembrinella randa* Walker, 1849	1	1	1	–	3
*Eumesembrinella benoisti* Séguy, 1925			1		1
*Huascaromusca aeneiventris* Wiedemann, 1830	–	3	–	–	3
*Total*	**623**	**661**	**263**	**1,279**	**2,826**

Table 2 showed the correlations between the abiotic variables and the abundance of individuals of Calliphoridae and Mesembrinellidae, where all variables had higher values during summer and autumn, while the lowest values were present between winter and spring.

**Table 2:** Relationship between the abiotic factors of minimum, average, and maximum temperatures in ºC, relative humidity in %, and rainfall in mm; of each season of the year on the total abundance of Calliphoridae and Mesembrinellidae collected summing the three environments: forest area (Parque Estadual dos Três Picos - Cachoeiras de Macacu), rural area (UFRRJ - Seropédica), and urban area (UNIRIO, Urca campus - RJ), between 2021 and 2022.

Season	Total AbundanceParte superior do formulário	Minimum Temperature	Average Temperature	Maximum TemperatureParte superior do formulárioParte inferior do formulário	Relative Humidity	Rainfall
Autumn	623	16,94	20,32	25,12	81,25	2,93
Winter	661	14,76	18,73	24,20	77,6	2,83
Spring	258	15,32	19,29	24,47	78,55	2,45
Summer	1279	19,74	22,28	26,80	82,84	14,51

Table 3 showed the Spearman correlation between the abiotic factors described above and the abundance of species collected from the two families. With a significance level set at 0.05, the vast majority of species did not show significant correlation between their respective abundances and abiotic factors. The Spearman rho values that measure the strength of the association between two variables, where in -1 the correlation was strongly negative, and in 1 the correlation was strongly positive were only analyzed when the p value was significant, which occurred only for the species *L. eximia* and *M. bellardiana*. Additionally, some species did not have a sufficient sample size to be analyzed.

**Table 3:** Spearman’s correlation between abiotic factors (temperature, relative humidity and precipitation) and the abundance of species collected from Calliphoridae and Mesembrinellidae in three environments: the forest area (Parque Estadual dos Três Picos- Cachoeiras de Macacu), rural area (UFRRJ- Seropédica) and urban area (UNIRIO, campus Urca- RJ) across the four seasons of the year between 2021 and 2022.

Taxa	Temperature		Relative Humidity		Rainfall	
	p	rho	p	rho	p	rho
CALLIPHORIDAE						
*Chrysomya albiceps*	0.76	-0.23	0.76	-0.23	0.76	-0.23
*Chrysomya megacephala*	0.58	0.22	0.50	0.27	0.29	0.42
*Cochliomyia hominivorax*	0.81	0.12	0.13	-0.67	0.13	0.67
*Hemilucilia benoisti*	0.22	-0.50	1	0	0.22	-0.58
*Hemilucilia segmentaria*	0.81	0.05	0.56	0.13	0.43	0.18
*Hemilucilia semidiaphana*	0.94	-0.22	0.46	0.23	0.10	0.48
*Lucilia cuprina*	0.06	0.78	0.81	0.12	0.08	0.35
*Lucilia eximia*	0.01	-0.50	0.28	0.22	0.08	0.35
*Paralucilia nigrofacialis*	–	–	–	–	–	–
MESEMBRINELLIDAE						
*Laneela nigripes*	0.33	0.39	0.90	0.04	1	0
*Mesembrinella bellardiana*	0.56	-0.24	0.21	-0.48	0.03	-0.73
*Mesembrinella peregrina*	–	–	–	–	–	–
*Mesembrinella semihyalina*	0.42	-0.57	0.42	-0.57	0.42	-0.57
*Mesembrinella currani*	0.42	-0.57	0.42	-0.57	0.42	-0.57
*Eumesembrinella quadrilineata*	1	0	0.71	0.19	1	0
*Eumesembrinella cyaneicyncta*	–	–	–	–	–	–
*Eumesembrinella randa*	1	0	1	0	1	0
*Eumesembrinella benoisti*	–	–	–	–	–	–
*Huascaromusca aeneiventris*	–	–	–	–	–	–

**Fig 3:**
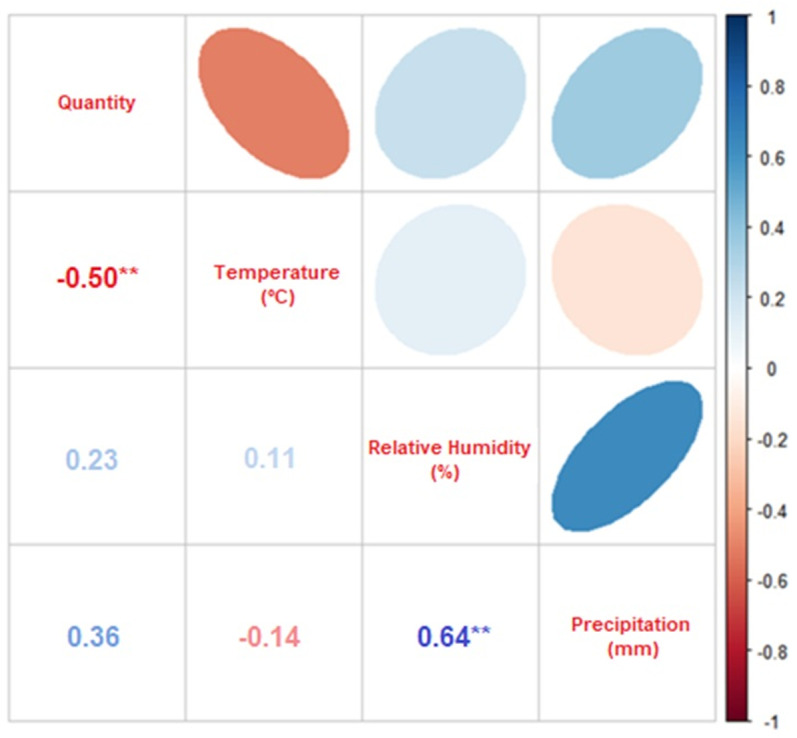
Spearman’s correlation between the abundance of *Lucilia eximia* and the abiotic factors of mean temperature, relative humidity, and rainfall index. Correlations marked with ** are significant at the 0.01 level.

When the Spearman linear correlation plot shows a circle tending toward an ellipse formation it was indicative that there was a correlation; and the more elliptical the circle, the more significant the correlation. The bluer the color of the circle, the closer the value was to 1, indicating a strongly positive correlation; similarly, the redder the circle, the closer the value was to -1, indicating a strongly negative correlation. Therefore, in Fig 3 there was a negative correlation between temperature and abundance of *L. eximia,* represented by the first circle from left to right of the first line, which was presented in a reddish ellipse shape. Meanwhile, the other two circles of this line, which represented the correlations between abundance and relative humidity of the air, and abundance and precipitation, their shape had little elliptical shape and in light blue coloration, indicating no correlation between abundance and these abiotic factors.

**Fig 4:**
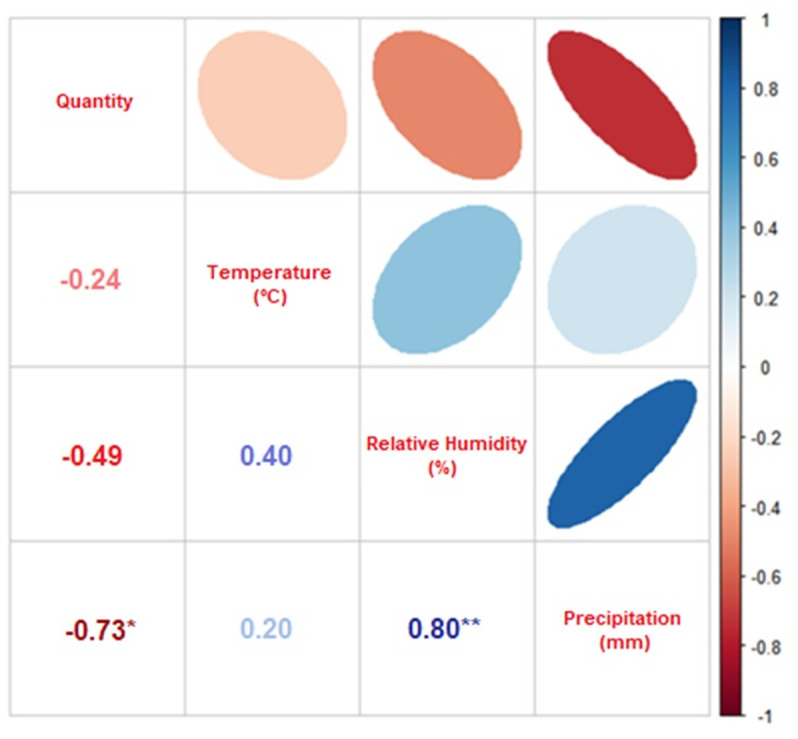
Spearman’s correlation between the abundance of *Mesembrinella bellardiana* and the abiotic factors of mean temperature, relative humidity, and rainfall index. Correlations marked with *  are significant at the 0.05 level, and marked with **are significant at the 0.01 level.

The Spearman correlation plot that evaluates the *M. bellardiana* species indicated in Fig 4 that there was a strongly negative correlation between precipitation and abundance, as the third circle from left to right of the first line had a strong elliptical shape and an intense red coloration. For the other abiotic factors of temperature and relative humidity of the air, the circles exhibited a less pronounced elliptical shape and lighter color, indicating that there was no correlation between these factors and the abundance of the species.

**Fig 5:**
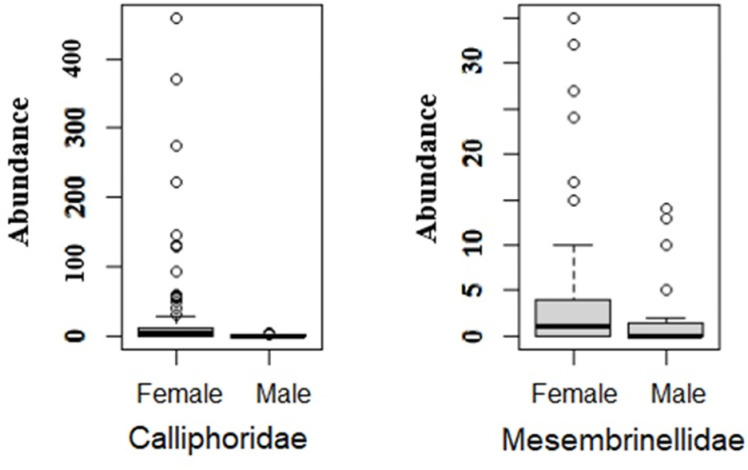
*Box plot* of the abundance of female and male individuals collected from the Calliphoridae and Mesembrinellidae families in a forest area (Parque Estadual dos Três Picos- Cachoeiras de Macacu), rural area (UFRRJ- Seropédica) and urban area (UNIRIO, campus Urca- RJ) during 2021 and 2022.

The *Box plot* of the abundance of female and male individuals collected from the two families, being analyzed separately, was represented in Fig 5, where the the Wilcoxon test revealed a significant higher abundance of females in the captures for both families. However, the difference was statistically greater within the family Calliphoridae (W = 60.49, p = 5.844x10^-12^) compared to the family Mesembrinellidae (W = 1231.5, p = 0.019). Consequently, the difference between the total number of females and males was smaller in this second family in relation to the first.

## Discussion

The main abiotic factor that affects seasonal variations in the occurrence of flies is the temperature of the environment during sampling [19], and should always be analyzed in order to know the activities of these flies so important from the medical, ecological and forensic perspective [20]. However, contrary to the results obtained by Azevedo and Krüger [21], abiotic factors in this study did not significantly influence the temporal dynamics of the families Calliphoridae and Mesembrinellidae that occurred throughout the four seasons of the year. Gadelha et al. [22] demonstrated that the main abiotic factors that influence reproductive biology, larval development and dispersion of dipterans are mainly temperature, then relative humidity and then precipitation. For this last factor, the intensity of precipitation should be taken into account, as heavy and/or prolonged rains can soak the soil, making the pupae unviable. For Sontigun et al. [23] the capture of calliphorids is positively correlated with temperature, negatively with relative humidity, and without correlation with precipitation; however in other studies [24,25], the same abiotic factors did not significantly influence the richness and abundance of Calliphoridae; except in this present study, which has evidenced a negative correlation between temperature and abundance for *L. eximia* and strongly negative correlation between precipitation and abundance for *M. bellardiana*; indicating that these species had an aversion to the increase in temperature and precipitation, respectively at the collection sites.

Species of the genus *Chrysomya* occurred only in the winter and summer seasons; with a total number of individuals smaller than the species of the genera *Lucilia* and *Hemilucilia*; the first occurring in all seasons except *L. cuprina* which was not present during the winter; while *H. segmentaria* and *H. semidiaphana* occurred in all seasons*, H. benoisti* were present only during the autumn and summer. According to Dufek et al. [24] also recorded lower occurrence of Calliphoridae in the winter months. *Cochliomyia hominivorax* did not appear during autumn, but its occurrence is very much related to the number of animals that serve as hosts for their larvae, which are biontophagous, differently from all other species collected [26]. *Paralucilia nigrofacialis* were the only species that occurred only in the summer. The species of the family Mesembrinellidae occurred in smaller numbers and with occasional appearances in different seasons of the year, except for the species *La. nigripes* and *M. bellardiana,* which were present in all seasons of the year and represented the most abundant species collected from this family. According to Williams and Villet [27] abiotic factors influence the abundance of Calliphoridae regardless of the season.

In general, several species showed higher abundance during the summer, except *H. segmentaria, H. benoisti* and *M. bellardiana* which had higher abundances during the autumn and *M. peregrina* which appeared only in winter. This data corroborates Babcock et al. [28] whose sampling was larger during the summer. *Chrysomya albiceps, M. semihyalina, M. currani, E. quadrilineata, E. cyaneicyncta, E. randa, E. benoisti,* and *Hu. aeneiventris* did not present higher abundance during the summer, however, in no season these species obtained a higher number than three specimens collected, indicating that they present low populations in the three areas of collection or aversion to the type of bait used for capture, the bovine liver.

Several studies agree that Calliphoridae has a tendency of more females than males collected in different types of attractive baits, locations, altitudes or times of the year [29]. For Sontigun et al. [23] the number of females exceeds that of males by a ratio of 2.36 to 1; while in this study the total number of females collected was more than twenty times greater than the total number of males. According to Dufek et al. [30] there was a higher probability of collecting more males from different calliphorid species in more anthropic environments compared to the more natural environment; while for this study the total number of males was significantly lower than the total number of females both in the three collection environments and during the four seasons of the year, but for species of the family Mesembrinellidae, which are exclusively found in natural environments [31], the ratio of females to males is lower when compared to the family Calliphoridae. This is a fact that should be carefully analyzed in future studies, as the lower sex ratio difference could be explained by the fact that the substrate used by the larval phase of mesembrinellids, after partially developing inside the maternal oviduct, is still poorly understood; and additionally, Mesembrinellidae oviposition is much less abundant compared to Calliphoridae [12].

As evidenced in the literature the aggregation of competitors in environments with spatial heterogeneity, including ephemeral substrates such as carcasses, can influence the coexistence of multiple species [32,33]. Therefore, intra- and interspecific competition within these necrophagous trophic guilds represents a complex phenomenon that directly impacts the population dynamics of different species. Observations from this study support this idea, showing that regardless of the season, more than half of the 19 collected species occurred in each season. Understanding the process of necrophagous insect arrival on available carcasses across different areas and seasons, where abiotic factors vary, is essential for forensic entomology. In the study performed by Moura et al. [34] was revealed a pattern of species arrival with aggregation in the early decomposition stages, similar to what was observed in this study, where traps remained exposed for 48 hours and none of the observed abiotic factors significantly influenced species abundance or aggregation from both studied families as observed by Shih-Tsai and Shiuh-Feng [35].

According to the research conducted by Greenberg and Tantawi [36] which was investigated the developmental rates of different Calliphoridae species under conditions of wide temperature variation, ranging from 12.5°C to 35°C, it was found that each species prefers a specific temperature range for larval development. However, in this present study, it’s important to note that throughout the four seasons, the abiotic factors did not vary significantly. The difference between minimum and maximum temperatures did not exceed 10°C, the fluctuation in relative humidity was less than 6%, and rainfall was below three milliliters in all seasons except summer, when it reached 14.51 mm. These findings suggest that the lack of statistical effect observed here may be due to the stability of abiotic factors across the three ecological collection areas, indicating that species from the families Calliphoridae and Mesembrinellidae may be less sensitive to these minor variations in abiotic factors compared to environments with more pronounced seasonal fluctuations, as demonstrated in the previously mentioned study.

In addition to the observed abiotic variables there is another factor that has potentially altered the distribution and abundance of the collected species that must be taken into account: the Covid-19 pandemic. Due to the restrictions maintained during the collection period between 2021 and early 2022, some factors that make an environment more anthropic were not in their standard compliance. Examples include lower rate of circulation of pedestrians and vehicles, closed commercial establishments causing less pollution and production of garbage exposed on the streets, affecting at first in part the distribution, abundance and richness of dipterans mainly in the urban environment [37]. Studies on the temporal and sexual fluctuations in different environments of occurrence of these species of flies are of wide relevance, since they offer important information about different ecological aspects, such as their bionomy, distribution and abundance, which assist in policies to control these insects, information on the state of preservation of environments, and in data that assist medical and forensic entomology.

Subsequent studies are expected to compare the attractiveness of baits between different species as well as between females and males, mainly in the Mesembrinellidae family. Further research is also needed in comparatively distinct ecological areas during temporal gradient in order to aid in a broader understanding of the temporal dynamics of the families Calliphoridae and Mesembrinellidae.

## Supporting information

S1 Data The data used in this study are available in the supplementary Excel file, which can be accessed via the following link: https://docs.google.com/spreadsheets/d/1_o50zvJsrXKqQkQKg3VdeB26Fr1sOgkb/edit?usp=sharing&ouid=107611510332645212094&rtpof=true&sd=true. This file contains the complete dataset referenced in the manuscript, organized by categories according to the methodology described.
(XLSX)

## References

[1] Tabarelli M, Pinto LP, Silva JMC, Hirota M, Bedê L. Challenges and Opportunities for biodiversity conservation in the brazilian atlantic forest. Conserv. Biol. 2005;19(3):695–700. doi: 10.1111/j.1523-1739.2005.00694.x

[2] Amorim D, Papavero N, Silva V, Lamas C, Nihei S, Ribeiro G, et al. A large scale survey of the Diptera of the Atlantic Forest. In International Congress of Dipterology, 7º. Abstract. Costa Rica, San José. 2010; 20–1.

[3] Couri M, Nessimian J, Mejdalani G, Monnè M, Lopes S, Mendonça M. Levantamento dos insetos da Mata Atlântica do Estado do Rio de Janeiro. Arq Museu Nacional. 2009.;67(3–4):3–4.

[4] Braack LEO. Community dynamics of carrion-attendant arthropods in tropical african woodland. Oecologia. 1987;72(3):402–9. doi: 10.1007/BF00377571 28311137

[5] Barton PS, Cunningham SA, Lindenmayer DB, Manning AD. The role of carrion in maintaining biodiversity and ecological processes in terrestrial ecosystems. Oecologia. 2013;171(4):761–72. doi: 10.1007/s00442-012-2460-3 23007807

[6] Kosmann C. Calliphoridae (Diptera): identificação, sinantropia e analise microbiológica. Tese (Doutorado em Biologia Animal). Instituto de Ciências Biológicas, Universidade de Brasília. 2013; 234 f.

[7] Maldonado MA, Centeno N. Quantifying the potential pathogens transmission of the blowflies (Diptera: Calliphoridae). Mem Inst Oswaldo Cruz. 2003;98(2):213–6. doi: 10.1590/s0074-02762003000200008 12764436

[8] d’Almeida JM, Lopes HDS. Sinantropia de dípteros caliptrados (Calliphoridae) no Estado do Rio de Janeiro. Arquivos da Universidade Federal Rural do Rio de Janeiro. 1983;6(1):31–8.

[9] Greenberg B. Flies as forensic indicators. J Med Entomol. 1991;28(5):565–77. doi: 10.1093/jmedent/28.5.565 1941921

[10] Tomberlin JK, Mohr R, Benbow ME, Tarone AM, VanLaerhoven S. A roadmap for bridging basic and applied research in forensic entomology. Annu Rev Entomol. 2011;56 401–21. doi: 10.1146/annurev-ento-051710-103143 20822449

[11] Guimaraes J. A systematic revision of the Mesembrinellidae, stat. nov. (Diptera, Cyclorrhapha). Arquivos de Zoologia. 1977;29(1):1–109.

[12] Whitworth TL, Yusseff-Vanegas S. A revision of the genera and species of the Neotropical family Mesembrinellidae (Diptera: Oestroidea). Zootaxa. 2019; 4659(1): 1–146. 10.11646/zootaxa.4659.1.1 31716728

[13] Mello R da S, Queiroz MMC, Aguiar-Coelho VM. Population fluctuations of calliphorid species (Diptera, Calliphoridae) in the Biological Reserve of Tinguá, state of Rio de Janeiro, Brazil. Iheringia, Sér Zool. 2007;97(4):481–5. doi: 10.1590/s0073-47212007000400019

[14] Weidner LM, Gemmellaro MD, Tomberlin JK, Hamilton GC. Evaluation of bait traps as a means to predict initial blow fly (Diptera: Calliphoridae) communities associated with decomposing swine remains in New Jersey, USA. Forensic Sci Int. 2017;278:95–100. doi: 10.1016/j.forsciint.2017.06.014 28710939

[15] Nunes MDP, Azevedo WT de A, da Silva AS, Lessa CSDS, Alencar J, Aguiar VM. Faunistic analysis of calliphoridae and mesembrinellidae (Diptera: Oestroidea) at different stages of bovine liver decomposition in the State of Rio de Janeiro. Life (Basel). 2023;13(9):1914. doi: 10.3390/life13091914 37763317 PMC10532495

[16] Mello RD. Chave para identificação das formas adultas das espécies da família Calliphoridae (Diptera, Brachycera, Cyclorrhapha) encontradas no Brasil. Entomologia e Vetores. 2003;10(2):255–68.

[17] Ayres M, Ayres Junior M, Ayres DL, Santos ADAD. BioEstat: aplicações estatísticas nas áreas das ciências biomédicas. Ong Mamiraua. Belém, PA. 2007. [Cited 2022 June 17]. Available from: http://www.mamiraua.org.br/ptbr/downloads/programas/

[18] Bussab WDO, Morettin PA. Estatística básica. In Estatística básica. Ed. Saraiva, São Paulo. 2010:xvi–540.

[19] Kökdener M, Polat E. Survey of forensically important Calliphoridae in Samsun. Bull. Leg. Med. 2016;21:67–71.

[20] Villet MHC, Clitheroe C, Williams KA.The temporal occurrence of flesh flies (Diptera, Sarcophagidae) at carrion-baited traps in Grahamstown, South Africa. African invertebrates.2017;58(1):1–8. doi: 10.3897/afrinvertebr.58.9537

[21] Azevedo RR, Krüger RF. The influence of temperature and humidity on abundance and richness of Calliphoridae (Diptera). Iheringia, Sér Zool. 2013;103(2):145–52. doi: 10.1590/s0073-47212013000200010

[22] Gadelha BQ, Ribeiro AC, Aguiar VM, Mello-Patiu CA. Edge effects on the blowfly fauna (Diptera, Calliphoridae) of the Tijuca National Park, Rio de Janeiro, Brazil. Braz J Biol. 2015;75(4):999–1007. doi: 10.1590/1519-6984.05614 26675918

[23] Sontigun N, Sukontason KL, Klong-Klaew T, Sanit S, Samerjai C, Somboon P, et al. Bionomics of the oriental latrine fly Chrysomya megacephala (Fabricius) (Diptera: Calliphoridae): temporal fluctuation and reproductive potential. Parasit Vectors. 2018;11(1)1–12. doi: 10.1186/s13071-018-2986-2 30005704 PMC6044043

[24] Dufek MI, Oscherov EB, Damborsky MP, Mulieri PR. Calliphoridae (Diptera) in Human-Transformed and Wild Habitats: Diversity and Seasonal Fluctuations in the Humid Chaco Ecoregion of South America. J Med Entomol. 2019;56(3):725–36. doi: 10.1093/jme/tjy234 30605537

[25] Alvarez Garcia DM, Pérez-Hérazo A, Amat E. Spatial and Temporal Variation of the Blowflies Community (Diptera: Calliphoridae) From an Urban Area in Northern South America. J Med Entomol. 2019;56(2):464–71. doi: 10.1093/jme/tjy211 30535268

[26] Guimarães J, Papavero N, Prado A. Miíases na região Neotropical. Revista Brasileira de Zoologia. 1983;1(4):239–416.

[27] Williams KA, Villet MH. Spatial and Seasonal Distribution of Forensically Important Blow Flies (Diptera: Calliphoridae) in Makhanda, Eastern Cape, South Africa. J Med Entomol. 2019;56(5):1231–8. doi: 10.1093/jme/tjz056 31081908

[28] Babcock NJ, Pechal JL, Benbow ME. Adult blow fly (Diptera: Calliphoridae) community structure across urban-rural landscapes in Michigan, United States. J Med Entomol. 2020;57(3):705–14. doi: 10.1093/jme/tjz246 31879776

[29] Mulieri PR, Torretta JP, Schnack JA, Mariluis JC. Calliphoridae (diptera) of the coastline of buenos aires, Argentina: species composition, numerical trends, and bait’s preferences. Entomological News. 2006;117(2):139–48. doi: 10.3157/0013-872x(2006)117[139:cdotco]2.0.co;2

[30] Dufek MI, Battán-Horenstein M, Mulieri PR. Blow flies, synanthropy and sex ratio: Are the deviations in the sex proportion linked to human transformation of landscapes?. Acta Trop. 2021;222:106052. doi: 10.1016/j.actatropica.2021.106052 34273305

[31] Gadelha B, Ferraz A, Coelho V. A importância dos mesembrinelíneos (Diptera: Calliphoridae) e seu potencial como indicadores de preservação ambiental. Oecologia Brasiliensis. 2009;13:660–5.

[32] Atkinson WD, Shorrocks B. Aggregation of larval diptera over discrete and ephemeral breeding sites: The implications for coexistence. Am. Nat. 1984;124(3):336–51. doi: 10.1086/284277

[33] Moura MO. Variação espacial como mecanismo promotor da coexistência em comunidades de insetos necrófagos. Rev Bras Zool. 2004;21(3):409–19. doi: 10.1590/s0101-81752004000300001

[34] Moura MO, Monteiro-Filho EL de A, Carvalho CJB de. Heterotrophic succession in carrion arthropod assemblages. Braz arch biol technol. 2005;48(3):477–86. doi: 10.1590/s1516-89132005000300018

[35] Yang S-T, Shiao S-F. Oviposition preferences of two forensically important blow fly species, Chrysomya megacephala and C. rufifacies (Diptera: Calliphoridae), and implications for postmortem interval estimation. J Med Entomol. 2012;49(2):424–35. doi: 10.1603/me11133 22493863

[36] Greenberg B, Tantawi TI. Different developmental strategies in two boreal blow flies (Diptera: Calliphoridae). J Med Entomol. 1993;30(2):481–4. doi: 10.1093/jmedent/30.2.481 8459428

[37] Decker RA, Haltiwanger J. Business entry and exit in the COVID-19 pandemic: A preliminary look at official data. 2022. FEDS Notes, May 6. Available from: https://www.federalreserve.gov/econres/notes/feds-notes/business-entry-and-exit-in-the-covid-19-pandemic-a-preliminary-look-at-official-data-20220506.html.

